# An international standardization to study the clinical use of lung ultrasound to discriminate viral, bacterial and atypical pneumonia in children with community acquired pneumonia

**DOI:** 10.1186/s13052-025-02113-5

**Published:** 2025-09-24

**Authors:** Lorenzo Di Sarno, Mariantonietta Francavilla, Azzurra Orlandi, Rosa Morello, Cristina De Rose, Luca Tagliaferri, Anna Clemente, Maria Chiara Supino, Anna Maria Musolino, Danilo Buonsenso

**Affiliations:** 1 Department of Woman and Child Health and Public Health, Fondazione Policlinico Universitario A. Gemelli IRCCS, Rome, Italy; 2 Department of Radiology, Giovanni XXIII Children Hospital, Azienda Ospedaliero Universitaria Consorziale Policlinico, Bari, 70124 Italy; 3 Pediatric Infectious Diseases Unit, Giovanni XXIII Children Hospital, Azienda Ospedaliero Universitaria Consorziale Policlinico, Bari, 70124 Italy; 4 Dipartimento di Diagnostica per Immagini e Radioterapia Oncologica, Fondazione Policlinico Universitario “A. Gemelli” IRCCS, Rome, Italy; 5 Bambino Gesù Children’s Hospital IRCCS, Hospital University Pediatrics Clinical Area, Rome, Italy; 6 Dipartimento di Scienze della Vita e Sanità Pubblica, Area Pediatrica, Università Cattolica del Sacro Cuore, Largo Gemelli 8, Rome, 00168 Italy

## Abstract

**Background:**

Community-acquired pneumonia (CAP) is a leading cause of morbidity and mortality in the pediatric population worldwide. Establishing a definitive etiological diagnosis in children with CAP remains challenging, as clinical, laboratory, and radiologic findings are often insufficient. Consequently, empirical and frequently unnecessary antibiotic treatments are commonly prescribed.

**Main body:**

Lung ultrasound (LUS) has demonstrated diagnostic accuracy comparable to chest X-ray (CXR) for CAP, while eliminating radiation exposure. Emerging evidence suggests that LUS may also differentiate the underlying etiologies of CAP. Assuming that distinct CAP etiologies exhibit characteristic LUS features, we aim to design a study protocol to develop a predictive model that integrates a child’s clinical information with their specific LUS patterns to inform individualized treatment strategies.

**Conclusions:**

This clinical approach will comprehensively evaluate clinical, laboratory, LUS, and outcome data from pediatric patients with CAP of various causes. If more centers will use the same approach, this will allow to gather in the short time data from large and diverse cohorts to facilitate the optimization and understanding of how LUS can potentially help understanding the etiology of CAP. The integrated data will be used to support tailored management strategies for pediatric CAP.

**Supplementary Information:**

The online version contains supplementary material available at 10.1186/s13052-025-02113-5.

## Background

Community-acquired pneumonia(CAP) represents the single largest cause of death and morbidity in children worldwide [1]. Respiratory viruses are the most common cause of CAP in preschool children, followed by bacteria.

In the majority of patients, clinical manifestations frequently are nonspecific, thereby posing a clinical diagnostic challenge [2]. These common presentations can include a wide range of symptoms, including a persistent or intermittent cough, the presence of fever, tachypnea and dyspnea [2]. Consequently, relying solely on these clinical items may not be sufficient for a definitive etiological diagnosis. The British Thoracic Society guidelines offer more targeted criteria for suspicion of bacterial pneumonia [3]. These guidelines recommend considering a diagnosis of bacterial pneumonia in previously healthy children who present with the combination of a persistent fever, the physical finding of chest retractions and an elevated respiratory rate. CAP can be caused by viruses, bacteria, or co-infections, with causative agents varying by age [3]. In the post-immunization era, viral pathogens constitute at least 70% of pediatric CAP cases, with prevalent agents including Respiratory Syncytial Virus (RSV), Rhinovirus, Influenza, Human Metapneumovirus, and Adenovirus, particularly affecting children under the age of five. Viral pneumonia represents the majority of CAP cases managed in outpatient settings [4]. Common bacterial pathogens include Streptococcus pneumoniae, Haemophilus influenzae, and Mycoplasma pneumoniae. The atypical bacteria Mycoplasma pneumoniae and Chlamydia pneumoniae are frequently implicated as causative agents of pneumonia in children older than five years [5].

Identifying the causative pathogen is critical, particularly in hospitalized children, as it leads to appropriate therapeutic decisions. However, establishing a etiological diagnosis for CAP in children remains challenging without invasive methods, and chest X-rays (CXR) have not been effective in determining CAP etiology [2, 6]. Clinical manifestations of bacterial, atypical bacterial, and viral pneumonia often overlap and cannot reliably differentiate between these causes, whereas laboratory markers, i.e., white blood cell count, C-reactive protein, and serum procalcitonin (which shows 85% sensitivity but only 45% specificity in excluding typical bacterial CAP), also lack diagnostic precision [7]. Consequently, children with CAP frequently receive unnecessary empirical antibiotic therapy, which contributes to the widespread development of antimicrobial resistance and increases the likelihood of potential adverse effects. Therefore, there is an urgent need for new methods that are fast, non-invasive, and easily accessible in outpatient settings to optimize and personalize the management of children with suspected CAP [8].

## Main text

Current guidelines primarily recommend a general approach to pediatric CAP and do not provide detailed treatment strategies [3, 9, 10]. Nonetheless, modern pediatric medicine necessitates the provision of bespoke, personalized treatments [11].

Recent evidence demonstrates that lung ultrasound (LUS) is highly accurate in diagnosing pediatric CAP and is not inferior to CXR [12–16]. LUS has demonstrated higher sensitivity (up to 97%) and comparable specificity for detecting CAP when compared to CXR, particularly excelling in the identification of small, subpleural consolidations and pleural effusions that may be missed by radiography [14]. Furthermore, LUS can be safely repeated for monitoring disease progression or response to therapy, which is valuable for ongoing clinical management. Additionally, LUS offers significant advantages including being radiation-free, low-cost, bedside-accessible, and suitable for repeated use, making it particularly advantageous in pediatric populations where minimizing radiation exposure is critical [17]. While chest computed tomography (CT) is regarded as the gold standard for identifying pulmonary lesions in pediatric CAP, attributable to its high sensitivity (frequently above 95%) and capacity to characterize intricate findings like parenchymal necrosis, cavitation, and subtle consolidations [18], it’s usually reserved for complex or unclear cases, not as a primary diagnostic tool like in other pediatric conditions [19]. This is largely due to significant concerns about radiation exposure in children. Despite its proven high accuracy, LUS as a diagnostic tool for pediatric CAP faces several limitations.

A primary concern is operator dependency, where the accuracy of LUS is heavily dependent on the sonographer’s expertise and experience. Variations in interpretation, especially by less skilled personnel lacking standardized training, can lead to misdiagnosis [20].

Technical constraints related to ultrasound equipment also impact image quality and diagnostic reliability. Factors such as probe type selection, time gain compensation, and beam focusing can lead to misdiagnosis of pulmonary consolidations.

Patient-related factors are furtherly challenging. Obesity, in particular, can impair ultrasound wave penetration due to increased chest wall thickness, thereby reducing image clarity and diagnostic yield [21]. Furthermore, the inability of some pediatric patients to remain still during the examination can hinder image acquisition and prolong the procedure, potentially compromising diagnostic accuracy [22].

Moreover, LUS has a limited ability to detect lesions not in direct contact with the pleura or located in lung areas difficult to access by ultrasound, such as deep parenchymal lesions, which may result in false negatives [23].

Early studies identified specific LUS patterns useful for diagnosing viral lower respiratory tract infections and bronchiolitis in children [24–28]. Recent works from the literature suggested LUS features can be used to determine the etiological cause of pediatric CAP [29]. In a prospective study by Copetti et al., distinct LUS features were identified for viral and bacterial pneumonia, with viral infections typically showing multiple, small subpleural consolidations and confluent B-lines, whereas bacterial pneumonia was characterized by larger consolidations with air bronchograms and pleural effusions [30]. Additionally, Caiulo et al. emphasized that LUS could reliably distinguish between bronchiolitis, viral pneumonia, and bacterial pneumonia based on the size and distribution of consolidations, the presence of pleural line abnormalities, and the characteristics of B-lines [31]. Berce evaluated 147 children hospitalized because of CAP, showing that LUS-detected consolidations in viral CAP were significantly smaller, with a median diameter of 15 mm, compared to 20 mm in atypical bacterial CAP(*p* = 0.05) and 30 mm in bacterial CAP(*p* < 0.001) [32]. Musolino et al. demonstrated that the presence of dynamic air bronchograms and larger consolidations were significantly associated with bacterial CAP, while viral CAP more often presented with interstitial patterns and smaller, patchy consolidations [33]. Buonsenso et al. showed that specific LUS patterns on diagnosis and after 48 h of treatments (bronchograms, consolidation size, characteristics of pleural effusion) were predictive of main aetiologic groups and of antibiotic response in children with CAP, more than clinical and laboratory data [34].

Furthermore, recent basic science and clinical studies are elucidating the understanding and genesis of artifacts resulting from the ultrasound-lung interaction [35]. In particular, by performing physical analyses on artificial models and using modern deep learning strategies to train a fully convolutional neural network, researchers showed that B-lines have different morphologies according to the medical conditions that generate them (interstitial lung diseases, cardiogenic and non-cardiogenic lung edema, interstitial pneumonia and lung contusion) [36–38]. Despite the presence of limited and preliminary data, a consensus classification integrating LUS findings with etiological agents has yet to be established within the scientific community. Indeed, more studies, hopefully prospective observational studies to collect more data on this regard, and later to be confirmed on randomized controlled trials, are highly needed. To do so, the scientific community would need to use a standardized approach for the specific use of LUS to discriminate bacterial, viral and atypical CAPs.

Provided that clinical data and inflammatory markers are accurately interpreted and systematically linked, LUS can contribute, via a stepwise approach, to a more comprehensive characterization of the etiological cause of CAP, thereby improving management strategies. With regard to this, the systematic and reproducible acquisition of a complete dataset—comprising clinical, laboratory, microbiology, and ultrasonomic parameters—is crucial for the establishment of a new paradigm of personalized medicine in the management of pediatric CAP. This paradigm would leverage the progressive phenotyping of individuals to tailor diagnostic and therapeutic interventions.

We have already procured promising data on how LUS can potentially be used as a clinical tool to optimize clinician’s discrimination of bacterial, viral and atypical CAPs, therefore sparing unnecessary antibiotics. Accordingly, the present manuscript aims to detail a fully structured protocol of our ongoing prospective multicenter investigation.

The aim of this study is to propose a standardized protocol for diagnosing pediatric CAP. This protocol focuses on identifying distinctive LUS features associated with specific etiologies of CAP in children and integrating these LUS findings with clinical and laboratory data. The goal is to enhance the accuracy of etiological diagnosis and to guide tailored treatment strategies.

Accordingly, this research seeks to develop a predictive model that combines LUS, clinical, and laboratory data to reliably differentiate among various etiologies of pediatric CAP. Furthermore, the study aims to establish a uniform model that can be adopted across multiple centers to ensure a consistent and standardized diagnostic approach.

## Materials and methods

### Study design

This is a prospective, observational study with the aim of investigating if there is an association between LUS features and the etiological diagnosis of CAP in the pediatric population.

### Ethical considerations

The study protocol should be submitted for review and approval by the Institutional Review Board or Ethics Committee at each participating site.

Participant selection will be conducted strictly according to the predefined inclusion and exclusion criteria to ensure the scientific validity and ethical integrity of the study. Informed consent will be obtained from parents or legal guardians prior to any study-related procedures, and assent will be sought from children as appropriate for their age and level of understanding, in accordance with the Declaration of Helsinki and local regulations.

To ensure robust data protection, all personal and health information will be handled in compliance with the European General Data Protection Regulation (GDPR) and relevant national laws. Each participant will be assigned a unique study identification code, and all data will be pseudonymized prior to analysis. Identifiable information will be accessible only to authorized study personnel at each site and will be stored on secure, password-protected servers. Data transfer between centers will use encrypted channels, and only de-identified datasets will be shared for central analysis. Access to the electronic database will be restricted to designated investigators, with audit trails maintained to monitor data access and modifications. All study documents and source data will be retained for the period required by regulatory authorities. Participants and their families will be informed of their rights regarding data access, correction, and withdrawal from the study at any time without consequences for their medical care.

### Study population and enrollment

#### Inclusion criteria

This protocol is currently proposed for children meeting all of the following criteria:

- Children with a clinical diagnosis of CAP having at least two of the following signs and symptoms: fever (> 38 °C), cough, dyspnea, abnormal auscultatory findings, with or without chest or abdominal pain.
- Only children who underwent LUS within six hours of the first clinical assessment and for whom clinical information on the outcome will be available.

#### Exclusion criteria

Children meeting any of the following criteria should be excluded by this study protocol:

- Patients with underlying diseases, including respiratory tract anomalies, immunodeficiency, cerebral palsy, neuromuscular diseases, congenital heart disease, and malignancy.
- Patients with other infection than CAP.
- Patients without parental consent.

### Enrollment procedure

Enrollment of eligible children in the study begins as soon as they present at a participating center and are identified by the attending physician. Once identified, the next crucial step is to obtain informed consent from their legal guardian. After consent is secured, comprehensive data collection is initiated using a standardized data collection form. A complete electronic registry composed of Electronic Health Records (EHR) collection forms will be created to minimize variability. The data collection form includes essential demographic information such as the child’s age, sex, weight, and height. In addition to demographics, a comprehensive medical history will be documented, covering any previous or current health conditions, medications, allergies, and prior hospitalizations that could influence the child’s current health status. Detailed information about the child’s presenting symptoms will also be recorded, including their onset, duration, severity, and any associated factors.

Following this, a clinical examination will be performed to specifically identify signs of respiratory conditions, such as the presence and character of crackles on lung auscultation. Other important physical findings like tachypnea, chest retractions, and nasal flaring will also be carefully noted. Beyond the initial clinical assessment, blood examinations will be performed to gather crucial laboratory data. These will include a complete blood count (CBC), a C-reactive protein (CRP) test, and a procalcitonin value. Additional blood tests may be conducted as deemed necessary by the attending physician, depending on the child’s presenting symptoms. A nasopharyngeal swab culture will be performed and its result will be recorded. Other microbiological data, including blood, pleural and bronchoalveolar lavage (BAL) cultures, will be recorded if available.

A specific section of the data registry will be dedicated to antibiotic therapy, including any antibiotics used prior to Pediatric Emergency Department (PED) admission and those administered from that point onward. Finally, the date and findings of the initial CXR will be meticulously documented, including both quantitative and qualitative information related to the radiological patterns identified.

A complete template of an EHR is shown in Supplementary Materials.

At this point, a comprehensive lung ultrasound evaluation will be performed in strict compliance with the acquisition protocol outlined below.

Seventy-two hours later, a second clinical, laboratory, and ultrasound evaluation of the patient will be conducted using the same criteria previously described. All collected data will be stored according to the previously specified procedures.

To ensure the reliability and reproducibility of LUS findings across all participating centers, rigorous standardization measures will be implemented. First, all operators will undergo a dedicated training program based on internationally recognized LUS protocols, including both theoretical instruction and hands-on supervised practice. Certification of proficiency will be required prior to independent scanning, and periodic refresher sessions will be organized to maintain high standards of performance. This detailed, standardized scanning protocol will be distributed to all centers, specifying probe selection, patient positioning, scanning windows, and image acquisition parameters. To minimize inter-operator variability, all LUS examinations will be performed using a predefined set of scanning planes and documented using a uniform image storage and labeling system, as detailed below. Centralized quality control will be established: a proportion of LUS images and clips from each center will be reviewed by an expert panel blinded to clinical data, ensuring adherence to protocol and consistency in interpretation. Additionally, all data will be entered into a centralized electronic database with built-in checks for completeness and consistency.

### LUS acquisition protocol

LUS should be performed using an ultrasound device that comply with the Medical Device Directive (MDD) 93/42/EEC and its subsequent amendments. The examination will be conducted within 6 h of the clinical diagnosis of CAP, without knowledge of microbiological, laboratory, or chest X-ray results. A linear probe (12–6 MHz) will be used for preschool children, while a curved probe (8–5 MHz) will be employed for older children if the visualization with the linear probe is not optimal. All ultrasound images will be systematically stored and securely archived in compliance with established professional guidelines to ensure their availability for clinical review, quality assurance, and medico-legal purposes. This comprehensive storage protocol will support clinical decision-making, facilitate quality control, and ensure compliance with regulatory requirements.

Fourteen areas (3 posterior, 2 lateral, and 2 anterior in each hemithorax) will be scanned per patient for 10 s according to a methodical scheme first described by Soldati et al. [39]. Scans need to be intercostal to cover the widest surface possible with a single scan.

A standard sequence of evaluations is proposed, using landmarks on chest anatomic lines. Echographic scans can be identified with progressive numbering starting from the right posterior basal regions. For a patient able to maintain the sitting position, the following areas are identified:

1. Right basal on the paravertebral line above the curtain sign;
2. Right middle on the paravertebral line at the inferior angle of the shoulder blade;
3. Right upper on the paravertebral line at the spine of the shoulder blade;
4. Left basal on the paravertebral line above the curtain sign;
5. Left middle on the paravertebral line at the inferior angle of the shoulder blade;
6. Left upper on the paravertebral line at the spine of the shoulder blade;
7. Right basal on the midaxillary line below the internipple line;
8. Right upper on the midaxillary line above the internipple line;
9. Left basal on the midaxillary line below the internipple line;
10. Left upper on the midaxillary line above the internipple line;
11. Right basal on the midclavicular line below the internipple line;
12. Right upper on the midclavicular line above the internipple line;
13. Left basal on the midclavicular line below the internipple line;
14. Left upper on the midclavicular line above the internipple line.

In cases of performance of LUS examinations in critical care settings (such as patients receiving invasive ventilation) and for patients who are not able to maintain the sitting position, the posterior areas might be difficult to evaluate. In these cases, the operator should try to have a partial view of the posterior basal areas, currently considered “hot areas” for pneumonia and start the echographic assessment from landmark number 7, where the operator will be at least able to detect any pleural effusions and characterize it.

The following ultrasound features will be systematically recorded:

- A comprehensive qualitative overview summarizing the LUS assessment, including A-lines, short vertical artifacts, multiple B-lines, white lung, subpleural consolidations, mixed consolidations, and multiple B-lines.
- The size of the primary lesion in each examined lung zone, generally defined as a subpleural lung parenchymal lesion (including consolidation and atelectasis).
- The presence and characteristics of bronchograms, specifying the type (air or fluid), morphology (arboriform or linear), position (deep if located more than 2 cm from the pleura, or superficial if adjacent to the pleura), and dynamicity during respiration (static or dynamic).
- The presence and characteristics of vertical artifacts or B-lines, including their length (short or long), distribution (spared or confluent), and position (unilateral or bilateral, perilesional or not).
- The presence and type of pleural effusion, classified as small or moderate/large, and characterized as simple (anechogenic and gravity-dependent) or complex (presence of septations, hyperechogenic spots, extending beyond the lung apex, not gravity-dependent, and potentially requiring drainage).

### Technical considerations for LUS

To optimize image quality and ensure reliable interpretation in LUS, several technical aspects must be carefully managed:

- Transducer selection: a linear transducer should be used whenever possible. If a linear transducer is not feasible due to the patient’s body habitus, a convex transducer should be selected based on the patient’s size to ensure adequate penetration and resolution.
- Focal point optimization: employ a single-focal point modality, avoiding multi-focusing techniques. Set the focal point on the pleural line to optimize the ultrasound beam shape for sensing the lung surface. Focusing on the pleural line concentrates the beam’s energy, resulting in improved detection of subtle lung surface details.
- Mechanical Index (MI) management: maintain a low MI, starting around 0.7 and reducing it further if visual findings permit. High MI values, particularly during prolonged observation, may pose a risk of lung tissue damage.
- Saturation artifact reduction: minimize saturation phenomena by carefully controlling the gain settings and, if necessary, reducing the mechanical index. Saturation occurs when the echo signal strength exceeds the capacity of the receiving electronics, distorting the signal and resulting in a loss of dynamic range. This manifests as areas of complete whiteout on the image, obscuring subtle variations in tissue response.
- Imaging mode restrictions: avoid cosmetic filters and specialized imaging modalities, such as harmonic imaging, contrast enhancement, Doppler, and compounding, as these can introduce artifacts or obscure relevant diagnostic information.
- Frame rate maximization: achieve the highest possible frame rate by disabling persistence and multi-focusing. A high frame rate improves temporal resolution, allowing for more accurate assessment of dynamic lung processes such as pleural sliding and the movement of artifacts.
- Standardized image archival: images should be saved in a standardized digital format, such as DICOM (Digital Imaging and Communications in Medicine), which facilitates integration with the hospital’s Picture Archiving and Communications System (PACS). If DICOM is not available, LUS data should be saved directly as a video format to capture dynamic lung characteristics. Visual findings, especially subtle changes, may not be apparent in every frame; therefore, acquiring short video clips allows for continuous monitoring of the lung surface beneath the landmark during respiratory cycles. This approach ensures that images are permanently retained for a minimum of three to five years, consistent with national recommendations, and are easily retrievable for multidisciplinary team discussions, follow-up assessments, and educational use. Each stored image should be clearly labeled with essential patient identifiers, examination date and time, facility information, anatomical location, and image orientation to maintain accuracy and traceability. All LUS examinations will be performed by the same physician at each center to reduce inter-operator variability and enhance consistency in image acquisition and interpretation. Confidentiality and data protection standards will be strictly observed throughout the storage and handling process.

### Scoring procedures

In the context of specific clinical conditions such as COVID-19, bronchiolitis, and neonatal respiratory distress syndrome, relatively simple scoring systems based on a limited number of LUS features have been successfully implemented. These scoring systems are feasible due to the homogeneous nature of LUS patterns observed in these cohorts of patients (e.g. normal or disrupted pleural lines, small subpleural consolidations, and white lung appearance). However, pediatric pneumonia is more challenging due to several reasons. First, there is considerable variability in the clinical phenotype, influenced by factors such as the patient’s age, immune response, and the causative pathogen. Additionally, the radiologic presentation of pneumonia varies significantly between patients, including differences in consolidation size, distribution, and the presence or absence of pleural effusion along with its characteristics. Furthermore, LUS enables detailed evaluation within consolidations, such as identifying the presence and type of bronchograms and hypoechoic areas. Consequently, a comprehensive LUS assessment of children with pneumonia is inherently more complex than the traditional LUS semeiotics used in other conditions. For these reasons, we have developed a more detailed LUS approach for children with suspected CAP. We believe this method should be more widely adopted by other centers to promote more reproducible and comparable research outcomes. Regardless of whether a specific scoring system is applied, the use of this detailed semeiotic approach is strongly recommended.

Several key parameters are assessed in the calculation of the lung ultrasound score, each contributing to the overall score according to defined criteria (Table 1), as detailed below:

A-lines, representing horizontal reverberations of the pleural line, are indicative of normal lung aeration and are assigned a score of 0. Short vertical artifacts (SVAs), defined as vertical artifacts not reaching the bottom of the screen at a 3 cm depth setting, are considered clinically insignificant in the context of suspected pneumonia and likewise receive a score of 0. A B line is a vertical, hyperechogenic ultrasound artifact that extends from the pleural line to the bottom of the screen without attenuation. Multiple B-lines—characterized by lines spaced less than 0.5 cm apart but still distinguishable—are pathological and assigned a score of 1, while confluent B-lines (indistinguishable from each other) are scored as 2. The white lung pattern, defined as a subpleural field with various shades of white in which individual B-lines cannot be distinguished, is a pathological finding and receives a score of 3.

Subpleural consolidations are evaluated both qualitatively and quantitatively. The mere presence of consolidation is scored as 4, while its size is further categorized: less than 1 cm in depth (score 0), 1–3 cm (score 1), and greater than 3 cm (score 2). The location is also considered, with bilateral consolidations scored as 1 and unilateral as 2. Bronchograms within consolidations are assessed for the presence and type: air bronchograms (score 1 for static/dynamic/superficial, score 2 for deep) and fluid bronchograms (score 1 if present).

Pleural effusions are classified by size and complexity: absent (score 0), small (score 0.5), moderate/large (score 1), with further distinction between simple effusions (anechoic, gravity-dependent, score 1) and complex effusions (with septations, hyperechoic spots, or non-gravity-dependent, score 2).

This structured scoring system enables a nuanced and reproducible assessment of CAP in children, facilitating both clinical decision-making and research comparability.

**Table 1:**
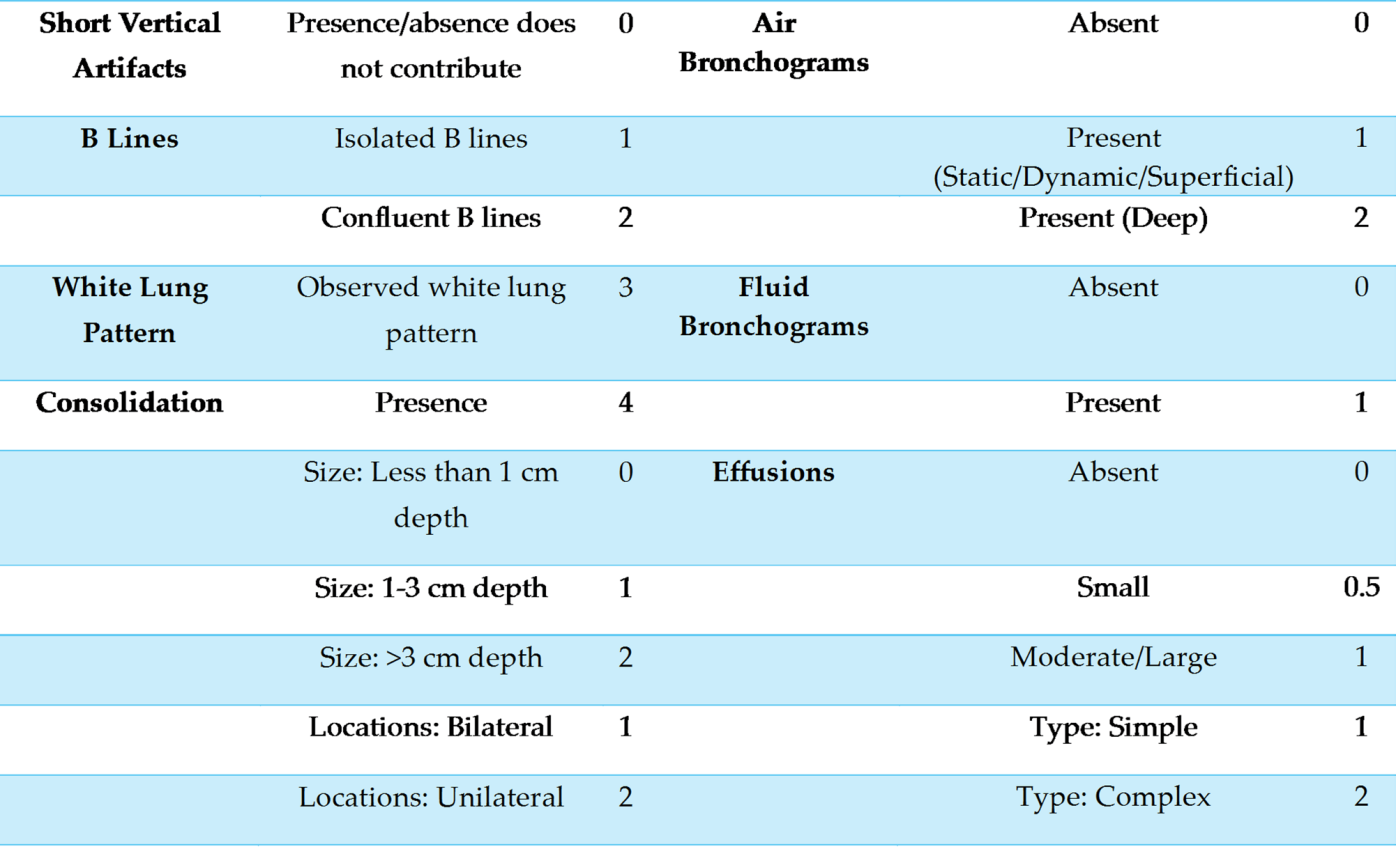
LUS scoring system, assigning points to specific sonographic features based on their presence and severity in pediatric CAP

LUS scoring system, assigning points to specific sonographic features based on their presence and severity in pediatric CAP.Etiological Classification of CAP

For this study, patients presenting with CAP will be systematically categorized based on their most probable microbial etiology. This classification process will involve a rigorous, multi-faceted assessment. A panel of three infectious disease specialists, blinded to patient discharge summaries, will review the final dataset to assign an etiological diagnosis using the criteria outlined below.

**Fig. 1:**
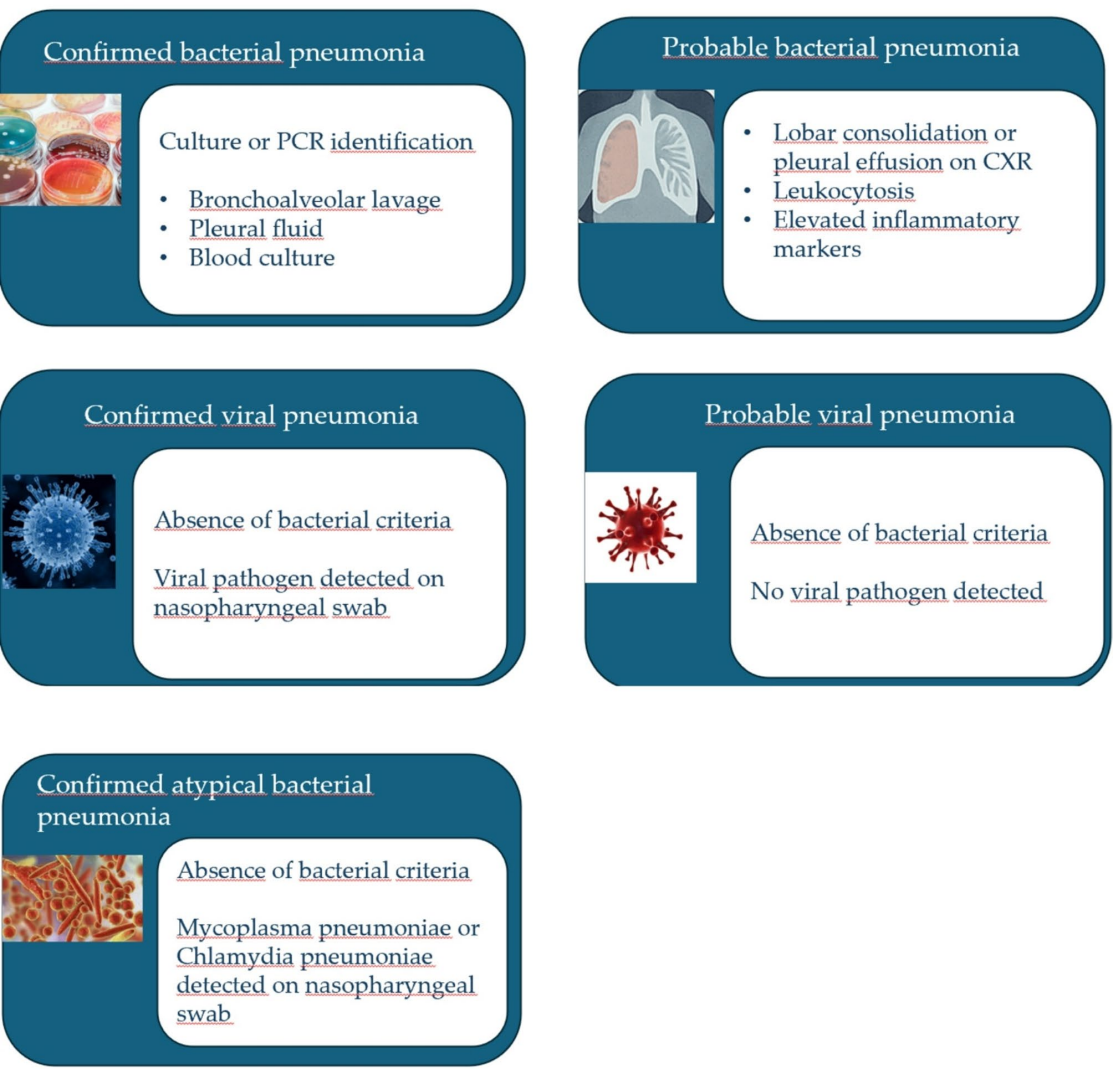
An overview of the etiological stratification strategy

A diagnosis of bacterial pneumonia will be established when a bacterial pathogen is definitively identified through culture or PCR-based methods in clinically relevant samples (e.g., bronchoalveolar lavage, pleural fluid, or blood cultures). These cases will be designated as “confirmed bacterial pneumonia.” In instances where direct microbiological confirmation is unavailable, but the patient exhibits a suggestive profile, a “probable bacterial pneumonia” diagnosis will be assigned. This will require the presence of at least two of the following: lobar consolidation or significant pleural effusion on CXR, leukocytosis (white blood cell count > 15 × 10^9^/L), or elevated inflammatory markers (C-reactive protein or procalcitonin). This probable diagnosis will be made even if viral pathogens or atypical bacteria are detected in nasopharyngeal swabs, acknowledging the possibility of co-infection. Patients with a detected viral pathogen in nasopharyngeal swabs will be categorized into the “viral pneumonia” group only after careful exclusion of bacterial co-infection. This exclusion is based on a comprehensive review of clinical, laboratory, radiological, and microbiological data, in line with a recent proposal of classification [40]. If no viral pathogens are detected and the patients do not meet the criteria for bacterial pneumonia, they will be classified as having “probable viral pneumonia.” If *Mycoplasma pneumoniae* or *Chlamydia pneumoniae* are detected in the nasopharyngeal swab and the patients do not meet the criteria for bacterial pneumonia, they will be classified as having “confirmed atypical bacterial pneumonia.” A comprehensive representation of the etiological stratification strategy is depicted in Fig. 1.

### Statistical analysis

Descriptive statistics for the study population will be presented as absolute numbers and percentages for categorical variables, and as medians with interquartile ranges (IQR) for continuous variables. Associations between categorical variables will be assessed using Chi-Squared tests, while Student’s T-tests will be employed for continuous variables.

Logistic regression models will be developed to examine the outcomes of interest, specifically: “confirmed viral pneumonia”, “confirmed plus probable viral pneumonia”, “confirmed bacterial pneumonia”, “confirmed plus probable bacterial pneumonia”, and “confirmed atypical bacterial pneumonia”. A hierarchical, multivariable logistic regression framework will be employed. Initial models will incorporate baseline clinical characteristics, followed by the sequential addition of laboratory parameters (including hematologic and microbiologic data) and radiological findings derived from conventional CXR or LUS.

For all models, CRP levels will be dichotomized into a binary variable, coded as 1 for CRP values below 40 mg/L and 0 for CRP values equal to or exceeding 40 mg/L. The incremental contribution of each data category to model performance will be assessed by monitoring changes in the Area Under the Receiver Operating Characteristic Curve (AUC), with an improvement observed as additional variables are incorporated beyond the clinical baseline.

Model calibration will be assessed using the Hosmer-Lemeshow goodness-of-fit test, and multicollinearity among predictors will be evaluated using the variance inflation factor. Effect size estimates will be reported as odds ratios with corresponding 95% confidence intervals. Sensitivity analyses will be conducted to assess the robustness of findings, including evaluating models in key subgroups and, where appropriate, using alternative CRP cut-off thresholds or excluding cases with missing data.

The equivalence of the ROC curves from the final models, one including LUS findings and the other based on conventional CXR, will be formally evaluated using DeLong’s test. All statistical analyses will be performed using Stata version 18.0 BE (StataCorp LLC, USA). Two-tailed hypothesis testing will be applied, with statistical significance defined as a p-value less than 0.05.

Sources of methodological bias and confounding will be systematically identified and addressed. Operator dependency, patient-related factors (i.e., body habitus and cooperation) and prior antibiotic use will be recorded and considered as possible confounders. When feasible, analyses will be stratified according to these variables to explore their impact on diagnostic accuracy. Where center variability may influence results, mixed-effects or hierarchical models will be employed. Any residual confounding or technical limitations impacting LUS interpretation will be transparently reported and discussed.

### Data standardization across different centers

To ensure data standardization across all participating centers, a unified electronic data capture (EDC) system will be implemented for clinical, laboratory, and imaging data. All centers will utilize a standardized EHR template, showed in **Supplementary Materials**, which defines required data fields, permissible values, and standardized terminology for clinical variables. This EHR template will be distributed to all sites and training will be provided to ensure consistent data entry practices. For LUS image storage, all centers will follow a harmonized protocol specifying image acquisition parameters, file formats (e.g., DICOM), and mandatory metadata (e.g., patient study ID, date, and anatomical site). LUS images and video clips will be uploaded to a secure, centralized, cloud-based repository with restricted access, allowing for real-time quality control and centralized review by an expert panel.

## Conclusions

By addressing the research questions, this study aims to develop a robust standardized framework of LUS evaluation to discriminate CAP etiology. Despite a notable epidemiological shift in childhood CAP, largely attributable to widespread pneumococcal conjugate and Haemophilus influenzae type B vaccinations, a concerningly high proportion of children with CAP continue to receive antibiotic prescriptions in both outpatient and inpatient settings [41, 42]. This pervasive practice signals significant challenges in accurate etiological diagnosis and prescribing practices, contributing to antibiotic resistance and potentially increasing healthcare utilization and adverse events. Consequently, the urgent need for enhanced diagnostic tools, targeted educational interventions, and robust antimicrobial stewardship programs is paramount to optimize antibiotic use in pediatric populations [43]. Clinical assessments, together with laboratory data and conventional radiological methods, have been demonstrated to be insufficient for establishing a direct and effective etiological diagnosis [44, 45] Even in a study where biomarkers showed a significant association with bacterial infection, establishing a reliable threshold remained elusive [46]. Recently, LUS has been established as an innovative, point-of-care diagnostic tool widely adopted across diverse pediatric clinical settings [47, 48]. As its integration into routine practice advances, LUS is emerging as a pivotal modality for etiological stratification in pediatric CAP, enhancing diagnostic precision and guiding management strategies [32, 34]. Given that similar LUS features can overlap across different etiologies and considering the limited evidence available—particularly from large-scale multicenter studies—a standardized and universally accepted classification system has yet to be established. Building upon these insights, this study protocol is designed to establish a standardized methodology for the comprehensive assessment of pediatric CAP, integrating clinical, laboratory, CXR, and LUS findings to build a progressively refined model for accurate etiological discrimination.

We strongly encourage the global scientific and medical community to collaborate in the creation and maintenance of a secure, protected, and internationally accessible database dedicated to pediatric CAP. This comprehensive repository would enable the standardized uploading and storage of multimodal imaging data—including radiographs, ultrasound scans, and CT images and videos—collected from children worldwide.

Such a centralized platform will significantly accelerate the development and validation of advanced, dedicated pattern recognition algorithms and artificial intelligence (AI) models designed to accurately detect and classify pathological findings specific to pediatric pneumonia [49, 50]. By facilitating large-scale data sharing and harmonization across diverse clinical settings and geographic regions, the database will enable robust cross-center comparisons and benchmarking, enhancing diagnostic consistency and reliability.

Moreover, this initiative will serve as a cornerstone for the expansion of telemedicine services, allowing real-time remote evaluation of imaging studies by expert clinicians, timely clinical advice, and collaborative case discussions that transcend geographical barriers. The platform will also support innovative tele-education and training programs, fostering continuous professional development through interactive teaching modules, image interpretation workshops, and AI-assisted learning tools [51].

Ultimately, the establishment of this international database will not only advance research and clinical care in pediatric pneumonia but also promote equitable access to state-of-art diagnostic resources and pave the way for future integration of AI-driven decision support systems in routine pediatric respiratory care.

## Supplementary Information

Below is the link to the electronic supplementary material.

Supplementary Material 1

Supplementary Material 2

## Data Availability

Not applicable.

## Abbreviations

CAP: Community acquired pneumonia
LUS: Lung ultrasound
RTI: Respiratory tract infections
LRTI: Low respiratory tract infections
CXR: Chest X ray

## References

[1] Rees CA, Kuppermann N, Florin TA. Community-Acquired Pneumonia in Children. Pediatr Emerg Care. 2023;39(12):968–976. 10.1097/PEC.0000000000003070. PMID: 38019716. 10.1097/PEC.0000000000003070 38019716

[2] Meyer Sauteur PM. Childhood community-acquired pneumonia. Eur J Pediatr. 2024;183(3):1129–36. 10.1007/s00431-023-05366-6. Epub 2023 Dec 19. PMID: 38112800; PMCID: PMC10950989. 38112800 10.1007/s00431-023-05366-6 PMC10950989

[3] Harris M, Clark J, Coote N, Fletcher P, Harnden A, McKean M, Thomson A, British Thoracic Society Standards of Care Committee. British Thoracic Society guidelines for the management of community acquired pneumonia in children: update 2011. Thorax. 2011;66 Suppl 2:ii1-23. 10.1136/thoraxjnl-2011-200598. PMID: 21903691. 10.1136/thoraxjnl-2011-200598 21903691

[4] Donà D, Brigadoi G, Grandinetti R, Pedretti L, Boscarino G, Barbieri E, Matera L, Mancino E, Bergamini M, Castelli Gattinara G, Chiappini E, Doria M, Galli L, Guarino A, Lo Vecchio A, Venturini E, Marseglia G, Verga MC, Di Mauro G, Principi N, Midulla F, Esposito S. Treatment of mild to moderate community-acquired pneumonia in previously healthy children: an Italian intersociety consensus (SIPPS-SIP-SITIP-FIMP-SIAIP-SIMRI-FIMMG-SIMG). Ital J Pediatr. 2024;50(1):217. 10.1186/s13052-024-01786-8. PMID: 39427174; PMCID: PMC11491012. 39427174 10.1186/s13052-024-01786-8 PMC11491012

[5] Chi J, Tang H, Wang F, Wang Y, Chen Z. Surge in Mycoplasma pneumoniae infection and respiratory viruses Co-infection in children with Community-Acquired pneumonia in the Post-Pandemic. Pediatr Health Med Ther. 2024;15:279–88. PMID: 39263589; PMCID: PMC11389692. 10.2147/PHMT.S473669 PMC11389692 39263589

[6] Korppi M, Don M, Valent F, Canciani M. The value of clinical features in differentiating between viral, Pneumococcal and atypical bacterial pneumonia in children. Acta Paediatr. 2008;97(7):943–7. 10.1111/j.1651-2227.2008.00789.x. Epub 2008 Apr 15. PMID: 18422803. 18422803 10.1111/j.1651-2227.2008.00789.x

[7] Ratageri VH, Panigatti P, Mukherjee A, Das RR, Goyal JP, Bhat JI, Vyas B, Lodha R, Singhal D, Kumar P, Singh K, Mahapatro S, Charoo BA, Kabra SK, Jat KR. Role of procalcitonin in diagnosis of community acquired pneumonia in children. BMC Pediatr. 2022;22(1):217. 10.1186/s12887-022-03286-2. PMID: 35443627; PMCID: PMC9020076. 35443627 10.1186/s12887-022-03286-2 PMC9020076

[8] Toro MS, Martínez JLV, Falcão RV, Prata-Barbosa A, Cunha AJLAD. Point-of-care ultrasound by the pediatrician in the diagnosis and follow-up of community-acquired pneumonia. J Pediatr (Rio J). 2021 Jan-Feb;97(1):13–21. Epub 2020 Aug 9. PMID: 32781037; PMCID: PMC9432299. 10.1016/j.jped.2020.07.003 PMC9432299 32781037

[9] Bradley JS, Byington CL, Shah SS, Alverson B, Carter ER, Harrison C, Kaplan SL, Mace SE, McCracken GH Jr, Moore MR, St Peter SD, Stockwell JA, Swanson JT. Pediatric infectious diseases society and the infectious diseases society of america. The management of community-acquired pneumonia in infants and children older than 3 months of age: clinical. Clin Infect Dis. 2011;53(7):e25–76. 10.1093/cid/cir531. practice guidelines by the Pediatric Infectious Diseases Society and the Infectious Diseases Society of AmericaEpub 2011 Aug 31. PMID: 21880587; PMCID: PMC7107838. 21880587 10.1093/cid/cir531 PMC7107838

[10] Michelow IC, Olsen K, Lozano J, Rollins NK, Duffy LB, Ziegler T, Kauppila J, Leinonen M, McCracken GH Jr. Epidemiology and clinical characteristics of community-acquired pneumonia in hospitalized children. Pediatrics. 2004;113(4):701-7. 10.1542/peds.113.4.701. PMID: 15060215. 10.1542/peds.113.4.701 15060215

[11] Chee E, Huang K, Haggie S, Britton PN. Systematic review of clinical practice guidelines on the management of community acquired pneumonia in children. Paediatr Respir Rev. 2022;42:59–68. 10.1016/j.prrv.2022.01.006. Epub 2022 Feb 4. PMID: 35210170. 35210170 10.1016/j.prrv.2022.01.006

[12] Buonsenso D, Tomà P, Scateni S, Curatola A, Morello R, Valentini P, Ferro V, D’Andrea ML, Pirozzi N, Musolino AM. Lung ultrasound findings in pediatric community-acquired pneumonia requiring surgical procedures: a two-center prospective study. Pediatr Radiol. 2020;50(11):1560–9. 10.1007/s00247-020-04750-w. Epub 2020 Aug 21. PMID: 32821992. 32821992 10.1007/s00247-020-04750-w

[13] Musolino AM, Tomà P, Supino MC, Scialanga B, Mesturino A, Scateni S, Battaglia M, Pirozzi N, Bock C, Buonsenso D. Lung ultrasound features of children with complicated and noncomplicated community acquired pneumonia: A prospective study. Pediatr Pulmonol. 2019;54(9):1479–86. 10.1002/ppul.24426. Epub 2019 Jul 1. PMID: 31264383. 31264383 10.1002/ppul.24426

[14] Yang Y, Wu Y, Zhao W. Comparison of lung ultrasound and chest radiography for detecting pneumonia in children: a systematic review and meta-analysis. Ital J Pediatr. 2024;50(1):12. 10.1186/s13052-024-01583-3. PMID: 38263086; PMCID: PMC10804756. 38263086 10.1186/s13052-024-01583-3 PMC10804756

[15] Yan JH, Yu N, Wang YH, Gao YB, Pan L. Lung ultrasound vs chest radiography in the diagnosis of children pneumonia: systematic evidence. Med (Baltim). 2020;99(50):e23671. 10.1097/MD.0000000000023671. PMID: 33327356; PMCID: PMC7738074. 10.1097/MD.0000000000023671 PMC7738074 33327356

[16] Bloise S, La Regina DP, Pepino D, Iovine E, Laudisa M, Di Mattia G, Nicolai A, Nenna R, Petrarca L, Mancino E, Frassanito A, Midulla F. Lung ultrasound compared to chest X-ray for the diagnosis of CAP in children. Pediatr Int. 2021;63(4):448–453. 10.1111/ped.14469. Epub 2021 Mar 4. PMID: 32935388. 10.1111/ped.14469 32935388

[17] Musolino AM, Di Sarno L, Buonsenso D, Murciano M, Chiaretti A, Boccuzzi E, Mesturino MA, Villani A. Use of POCUS for the assessment of dehydration in pediatric patients-a narrative review. Eur J Pediatr. 2024;183(3):1091–105. 10.1007/s00431-023-05394-2. Epub 2023 Dec 22. PMID: 38133810. 38133810 10.1007/s00431-023-05394-2

[18] Andronikou S, Goussard P, Sorantin E. Computed tomography in children with community-acquired pneumonia. Pediatr Radiol. 2017;47(11):1431–40. 10.1007/s00247-017-3891-0. Epub 2017 Sep 21. PMID: 29043419; PMCID: PMC5608781. 29043419 10.1007/s00247-017-3891-0 PMC5608781

[19] Shiroshita A, Kimura Y, Yamada A, Shirakawa C, Yue C, Suzuki H, Anan K, Sato K, Nakashima K, Takeshita M, Okuno T, Nitawaki T, Suzuki H, Igei H, Suzuki J, Tomii K, Ohgiya M, Kataoka Y. Prognostic Value of Computed Tomography in Empyema: A Multicenter Retrospective Cohort Study. Ann Am Thorac Soc. 2023;20(6):807–814. 10.1513/AnnalsATS.202210-868OC. PMID: 37166901. 10.1513/AnnalsATS.202210-868OC 37166901

[20] Pereda MA et al. Diagnostic accuracy of lung ultrasound performed by novice versus advanced sonographers for pneumonia in children: A systematic review and Meta-analysis. J Pediatr. 2019; [PMID: 31409764]. 10.1111/acem.13818 31211896

[21] Uppot RN. Technical challenges of imaging & image-guided interventions in obese patients. Br J Radiol. 2018;91(1089):20170931. 10.1259/bjr.20170931. Epub 2018 Jun 5. PMID: 29869898; PMCID: PMC6223172. 29869898 10.1259/bjr.20170931 PMC6223172

[22] Lin S. Procedural ultrasound in pediatric patients: techniques and tips for accuracy and safety. Pediatr Emerg Med Pract. 2016;13(6):1–38; quiz 24 – 5. Epub 2016 Jun 1. PMID: 27232771. 27232771

[23] Abid I, Qureshi N, Lategan N, Williams S, Shahid S. Point-of-care lung ultrasound in detecting pneumonia: A systematic review. Can J Respir Ther. 2024;60:37–48. 10.29390/001c.92182. PMID: 38299193; PMCID: PMC10830142. 38299193 10.29390/001c.92182 PMC10830142

[24] Gori L, Amendolea A, Buonsenso D, Salvadori S, Supino MC, Musolino AM, Adamoli P, Coco AD, Trobia GL, Biagi C, Lucherini M, Leonardi A, Limoli G, Giampietri M, Sciacca TV, Morello R, Tursi F, Soldati G, Ecobron Group. Prognostic role of lung ultrasound in children with bronchiolitis: multicentric prospective study. J Clin Med. 2022;11(14):4233. 10.3390/jcm11144233. PMID: 35887997; PMCID: PMC9316238. 35887997 10.3390/jcm11144233 PMC9316238

[25] La Regina DP, Bloise S, Pepino D, Iovine E, Laudisa M, Cristiani L, Nicolai A, Nenna R, Mancino E, Di Mattia G, Petrarca L, Matera L, Frassanito A, Midulla F. Lung ultrasound in bronchiolitis. Pediatr Pulmonol. 2021;56(1):234–9. 10.1002/ppul.25156. Epub 2020 Nov 24. PMID: 33151023. 33151023 10.1002/ppul.25156

[26] Di Mauro A, Ammirabile A, Quercia M, Panza R, Capozza M, Manzionna MM, Laforgia N. Acute bronchiolitis: is there a role for lung ultrasound?? Diagnostics (Basel). 2019;9(4):172. 10.3390/diagnostics9040172. PMID: 31683953; PMCID: PMC6963954. 10.3390/diagnostics9040172 PMC6963954 31683953

[27] Supino MC, Buonsenso D, Scateni S, Scialanga B, Mesturino MA, Bock C, Chiaretti A, Giglioni E, Reale A, Musolino AM. Point-of-care lung ultrasound in infants with bronchiolitis in the pediatric emergency department: a prospective study. Eur J Pediatr. 2019;178(5):623–32. 10.1007/s00431-019-03335-6. Epub 2019 Feb 12. PMID: 30747262. 30747262 10.1007/s00431-019-03335-6

[28] Buonsenso D, Supino MC, Giglioni E, Battaglia M, Mesturino A, Scateni S, Scialanga B, Reale A, Musolino AMC. Point of care diaphragm ultrasound in infants with bronchiolitis: A prospective study. Pediatr Pulmonol. 2018;53(6):778–86. 10.1002/ppul.23993. Epub 2018 Mar 26. PMID: 29578644. 29578644 10.1002/ppul.23993

[29] Stoicescu ER, Iacob R, Ilie AC, Iacob ER, Susa SR, Ghenciu LA, Constantinescu A, Cocolea DM, Oancea C, Manolescu DL. Differentiating viral from bacterial pneumonia in children: the diagnostic role of lung Ultrasound-A prospective observational study. Diagnostics (Basel). 2024;14(5):480. 10.3390/diagnostics14050480. PMID: 38472952; PMCID: PMC10931154. 38472952 10.3390/diagnostics14050480 PMC10931154

[30] Copetti R, Cattarossi L. Ultrasound diagnosis of pneumonia in children. Radiol Med. 2013;118(2):307–14. 10.1007/s11547-008-0247-8 18386121

[31] Caiulo VA, Gargani L, Caiulo S, et al. Lung ultrasound in bronchiolitis: comparison with chest X-ray. Eur J Pediatr. 2013;172(11):1649–56. 21468639 10.1007/s00431-011-1461-2

[32] Berce V, Tomazin M, Gorenjak M, Berce T, Lovrenčič B. The usefulness of lung ultrasound for the aetiological diagnosis of Community-Acquired pneumonia in children. Sci Rep. 2019;9(1):17957. 10.1038/s41598-019-54499-y. PMID: 31784642; PMCID: PMC6884636. 31784642 10.1038/s41598-019-54499-y PMC6884636

[33] Musolino AM, Supino MC, Buonsenso D, et al. Lung ultrasound features of children with complicated and non-complicated community acquired pneumonia: a prospective study. Pediatr Pulmonol. 2019;54(10):1479–86. 31264383 10.1002/ppul.24426

[34] Buonsenso D, Musolino A, Ferro V, De Rose C, Morello R, Ventola C, Liotti FM, De Sanctis R, Chiaretti A, Biasucci DG, Spanu T, Sanguinetti M, Valentini P. Role of lung ultrasound for the etiological diagnosis of acute lower respiratory tract infection (ALRTI) in children: a prospective study. J Ultrasound. 2022;25(2):185–97. 10.1007/s40477-021-00600-z. Epub 2021 Jun 19. PMID: 34146336; PMCID: PMC8213536. 34146336 10.1007/s40477-021-00600-z PMC8213536

[35] Soldati G, Demi M, Smargiassi A, Inchingolo R, Demi L. The role of ultrasound lung artifacts in the diagnosis of respiratory diseases. Expert Rev Respir Med. 2019;13(2):163–72. Epub 2019 Jan 10. PMID: 30616416. 30616416 10.1080/17476348.2019.1565997

[36] Demi M, Soldati G, Ramalli A. Lung ultrasound artifacts interpreted as pathology footprints. Diagnostics (Basel). 2023;13(6):1139. 10.3390/diagnostics13061139. PMID: 36980450; PMCID: PMC10047655. 36980450 10.3390/diagnostics13061139 PMC10047655

[37] Demi L, van Hoeve W, van Sloun RJG, Soldati G, Demi M. Determination of a potential quantitative measure of the state of the lung using lung ultrasound spectroscopy. Sci Rep. 2017;7(1):12746. 10.1038/s41598-017-13078-9. PMID: 28986558; PMCID: PMC5630606. 28986558 10.1038/s41598-017-13078-9 PMC5630606

[38] Arntfield R, VanBerlo B, Alaifan T, Phelps N, White M, Chaudhary R, Ho J, Wu D. Development of a convolutional neural network to differentiate among the etiology of similar appearing pathological B lines on lung ultrasound: a deep learning study. BMJ Open. 2021;11(3):e045120. 10.1136/bmjopen-2020-045120. PMID: 33674378; PMCID: PMC7939003. 33674378 10.1136/bmjopen-2020-045120 PMC7939003

[39] Soldati G, Smargiassi A, Inchingolo R, Buonsenso D, Perrone T, Briganti DF, Perlini S, Torri E, Mariani A, Mossolani EE, Tursi F, Mento F, Demi L. Proposal for international standardization of the use of lung ultrasound for patients with COVID-19: A simple, quantitative, reproducible method. J Ultrasound Med. 2020;39(7):1413–9. Epub 2020 Apr 13. PMID: 32227492; PMCID: PMC7228287. 32227492 10.1002/jum.15285 PMC7228287

[40] Nijman RG, Oostenbrink R, Moll HA, Casals-Pascual C, von Both U, Cunnington A, De T, Eleftheriou I, Emonts M, Fink C, van der Flier M, de Groot R, Kaforou M, Kohlmaier B, Kuijpers TW, Lim E, Maconochie IK, Paulus S, Martinon-Torres F, Pokorn M, Romaine ST, Calle IR, Schlapbach LJ, Smit FJ, Tsolia M, Usuf E, Wright VJ, Yeung S, Zavadska D, Zenz W, Levin M, Herberg JA, Carrol ED. PERFORM consortium (Personalized risk assessment in febrile children to optimize Real-life management across the European Union). A Novel Framework for Phenotyping Children With Suspected or Confirmed Infection for Future Biomarker Studies. Front Pediatr. 2021;9:688272. 10.3389/fped.2021.688272. PMID: 34395340; PMCID: PMC8356564. 34395340 10.3389/fped.2021.688272 PMC8356564

[41] van Houten CB, Naaktgeboren C, Buiteman BJM, van der Lee M, Klein A, Srugo I, Chistyakov I, de Waal W, Meijssen CB, Meijers PW, de Winter-de Groot KM, Wolfs TFW, Shachor-Meyouhas Y, Stein M, Sanders EAM, Bont LJ. Antibiotic Overuse in Children with Respiratory Syncytial Virus Lower Respiratory Tract Infection. Pediatr Infect Dis J. 2018;37(11):1077–1081. 10.1097/INF.0000000000001981. PMID: 29601448. 10.1097/INF.0000000000001981 29601448

[42] Ardillon A, Ramblière L, Kermorvant-Duchemin E, Sok T, Zo AZ, Diouf JB, Long P, Lach S, Sarr FD, Borand L, Cheysson F, Collard JM, Herindrainy P, de Lauzanne A, Vray M, Delarocque-Astagneau E, Guillemot D, Huynh BT, BIRDY study group. Inappropriate antibiotic prescribing and its determinants among outpatient children in 3 low- and middle-income countries: A multicentric community-based cohort study. PLoS Med. 2023;20(6):e1004211. 10.1371/journal.pmed.1004211. PMID: 37279198; PMCID: PMC10243627. 37279198 10.1371/journal.pmed.1004211 PMC10243627

[43] Kreitmeyr K, von Both U, Pecar A, Borde JP, Mikolajczyk R, Huebner J. Pediatric antibiotic stewardship: successful interventions to reduce broad-spectrum antibiotic use on general pediatric wards. Infection. 2017;45(4):493–504. 10.1007/s15010-017-1009-0. Epub 2017 Apr 10. PMID: 28397171. 28397171 10.1007/s15010-017-1009-0

[44] Heiskanen-Kosma T, Korppi M. Serum C-reactive protein cannot diferentiate bacterial and viral aetiology of communityacquired pneumonia in children in primary healthcare settings. Scand J Infect Dis. 2000;32(4):399–402. 10959648 10.1080/003655400750044971

[45] Flood RG, Badik J, Aronoff SC. The utility of serum C-reactive protein in differentiating bacterial from nonbacterial pneumonia in children: a meta-analysis of 1230 children. Pediatr Infect Dis J. 2008;27(2):95 – 9. 10.1097/INF.0b013e318157aced. PMID: 18174874. 10.1097/INF.0b013e318157aced 18174874

[46] Berg AS, Inchley CS, Fjaerli HO, Leegaard TM, Lindbaek M, Nakstad B. Clinical features and inflammatory markers in pediatric pneumonia: a prospective study. Eur J Pediatr. 2017;176(5):629–38. 10.1007/s00431-017-2887-y. Epub 2017 Mar 9. PMID: 28281094. 28281094 10.1007/s00431-017-2887-y

[47] Musolino AM, Tomà P, De Rose C, Pitaro E, Boccuzzi E, De Santis R, Morello R, Supino MC, Villani A, Valentini P, Buonsenso D. Ten years of pediatric lung ultrasound: A narrative review. Front Physiol. 2022;12:721951. PMID: 35069230; PMCID: PMC8770918. 35069230 10.3389/fphys.2021.721951 PMC8770918

[48] Musolino AM, Di Sarno L, Caroselli A, et al. Point-of-care ultrasound for the diagnosis of Swyer-James-MacLeod syndrome in pediatric emergency setting: a case report. Acta Biomed. 2024;95(6):e2024146. 10.23750/abm.v95i6.15840.

[49] Di Sarno L, Caroselli A, Tonin G, Graglia B, Pansini V, Causio FA, Gatto A, Chiaretti A. Artificial intelligence in pediatric emergency medicine: applications, challenges, and future perspectives. Biomedicines. 2024;12(6):1220. 10.3390/biomedicines12061220. PMID: 38927427; PMCID: PMC11200597. 38927427 10.3390/biomedicines12061220 PMC11200597

[50] Causio FA, DE Angelis L, Diedenhofen G, Talio A, Baglivo F. Workshop participants. Perspectives on AI use in medicine: views of the Italian society of artificial intelligence in medicine. J Prev Med Hyg. 2024;65(2):E285–9. 10.15167/2421-4248/jpmh2024.65.2.3261. PMID: 39430984; PMCID: PMC11487733. 39430984 10.15167/2421-4248/jpmh2024.65.2.3261 PMC11487733

[51] Suzuki R, Riley WJ, Bushman MS, Dong Y, Sekiguchi H. Tele-education in point-of-care ultrasound training. Ultrasound J. 2024;16(1):47. 10.1186/s13089-024-00394-1. PMID: 39466493; PMCID: PMC11519237. 39466493 10.1186/s13089-024-00394-1 PMC11519237

