# Supplementary material for: An international standardization to study the clinical use of lung ultrasound to discriminate viral, bacterial and atypical pneumonia in children with community acquired pneumonia
Source: Ital J Pediatr. 2025 Sep 24;51:269. doi: 10.1186/s13052-025-02113-5 (PMC12462357; doi:10.1186/s13052-025-02113-5)
Supplement: Supplementary file 1 — Supplementary Material 1 [file 13052_2025_2113_MOESM1_ESM.docx]

**Electronic Health Record (EHR)**

**Patient information**

| **Items** | **Details** | **Entry** |
| --- | --- | --- |
| Patient ID | Unique identifier for the patient |  |
| Date of Birth | Format: DD/MM/YYYY |  |
| Age | Automatically calculated from Date of Birth and Date of Assessment |  |
| Sex | ☐ Male ☐ Female |  |
| Ethnicity | ☐ Caucasian ☐ African ☐ Asian ☐ Hispanic ☐ Other |  |
| Weight (kg) | Measured weight at baseline |  |
| Height (cm) | Measured height at baseline |  |
| Body Mass Index (BMI) | Calculated BMI (kg/m²) |  |
| Primary Caregiver | Name and contact information |  |
| Residence | Urban / Suburban / Rural |  |
| Exposure to Tobacco Smoke | ☐ Yes ☐ No |  |
| Vaccination Status | ☐ Up to date ☐ Incomplete ☐ Unknown |  |
| Previous Hospitalizations | Number and reason for previous hospital admissions |  |
| Comorbidities | Check all that apply: |  |
|  | ☐ Asthma |  |
|  | ☐ Allergy |  |
|  | ☐ Other chronic respiratory conditions |  |
|  | ☐ Autoimmune diseases |  |
|  | ☐ Celiac disease |  |
|  | ☐ Genetic conditions |  |
|  | ☐ Other (specify) |  |
| Medications at Baseline | List any current medications (including inhalers, steroids, immunosuppressants, etc.) |  |
| Recent Infections | Any infections in the past 4 weeks (type and treatment) |  |
| Allergies | Document known allergies (drug, food, environmental) |  |
| Family History | Relevant family history of respiratory or autoimmune diseases |  |

**Clinical presentation**

| **Symptom/Sign** | **Baseline (0h)** | **72h Follow-up** |
| --- | --- | --- |
| Fever | ☐ Yes ☐ No | ☐ Yes ☐ No |
| T max (°C) |  |  |
| Days of fever |  |  |
| Cough | ☐ Yes ☐ No | ☐ Yes ☐ No |
| Rhinitis | ☐ Yes ☐ No | ☐ Yes ☐ No |
| Conjunctivitis | ☐ Yes ☐ No | ☐ Yes ☐ No |
| Respiratory sounds | ☐ Wheezing  ☐ Localized crackles/rales  ☐ Diffuse crackles/rales  ☐ Localized reduced ventilation  ☐ Bilateral reduced ventilation | ☐ Wheezing  ☐ Localized crackles/rales  ☐ Diffuse crackles/rales  ☐ Localized reduced ventilation  ☐ Bilateral reduced ventilation |
| Dyspnea | ☐ Yes ☐ No | ☐ Yes ☐ No |
| Respiratory rate |  |  |
| Nasal flaring | ☐ Yes ☐ No | ☐ Yes ☐ No |
| Chest retractions | ☐ Yes ☐ No | ☐ Yes ☐ No |
| Vomiting | ☐ Yes ☐ No | ☐ Yes ☐ No |
| Diarrhea | ☐ Yes ☐ No | ☐ Yes ☐ No |
| Abdominal pain | ☐ Yes ☐ No | ☐ Yes ☐ No |

**Laboratory findings**

| Parameter | Baseline (0h) | 72h Follow-up |
| --- | --- | --- |
| WBC (×10^9/L) |  |  |
| Neutrophils count (%) |  |  |
| Lymphocytes count (%) |  |  |
| Monocytes count (%) |  |  |
| C-Reactive protein (mg/L) |  |  |
| Procalcitonin (ng/mL) |  |  |

**Microbiology**

| **Test** | **Performed? (Y/N)** | **Result** |
| --- | --- | --- |
| Nasopharyngeal swab culture |  |  |
| Nasopharyngeal swab PCR |  |  |
| Blood culture |  |  |
| BAL culture |  |  |
| Urine pneumococcal antigen |  |  |
| Pleural fluid culture |  |  |

**Non-ultrasound imaging**

| **Imaging Modality** | **Performed? (Y/N)** | **Findings (check all that apply)** |
| --- | --- | --- |
| Chest X-ray |  | ☐ Interstitial pattern  ☐ Consolidation (☐ Monolateral ☐ Bilateral ☐ With air bronchogram)  ☐ Pleural effusion |
| CT scan |  | ☐ Interstitial pattern  ☐ Consolidation (☐ Monolateral ☐ Bilateral ☐ With air bronchogram)  ☐ Pleural effusion |

**Lung Ultrasound scoring**

| **US item** | **Baseline score** | **72h score** | **Notes** |
| --- | --- | --- | --- |
| -Multiple B lines |  |  | B lines with distance <0.5 cm, distinguishable, pathological (Score: 1) |
| - Confluent B lines |  |  | undistinguishable, pathological (Score: 2) |
| - White lung |  |  | Subpleural field with white shades, no distinct B lines, pathological (Score: 3) |
| **Consolidation:** |  |  |  |
| **-**Presence |  |  | (Score:4) |
| - Size of consolidation: |  |  | <1 cm depth (Score: 0), 1-3 cm depth (Score: 1), >3 cm depth (Score: 2) |
| - Location of consolidations |  |  | Bilateral (Score: 1), Monolateral (Score: 2) |
| **Air Bronchograms:** |  |  |  |
| - Absent |  |  | Score: 0 |
| - Present (Static) |  |  | Score: 1 |
| - Present (Dynamic) |  |  | Score: 1 |
| - Superficial (within 2 cm from pleural line) |  |  | Score: 1 |
| - Deep (> 2 cm from pleural line) |  |  | Score: 2 |
| **Fluid Bronchograms:** |  |  | Absent (Score: 0), Present (Score: 1) |
| **Effusion:** |  |  |  |
| - Effusion absent |  |  | Score: 0 |
| - Effusion present: small |  |  | Score: 0.5 |
| - Effusion present: moderate/large |  |  | Score: 1 |
| - Effusion type: simple |  |  | Score: 1 |
| - Effusion type: complex |  |  | Score: 2 |
| **Total Score** |  |  | Sum of all above scores |

**Therapy**

|  | **Drug Name** | **Total course length (days)** | **Days IV** | **Days Oral** |
| --- | --- | --- | --- | --- |
| Antibiotics (Oral) | 1: 2: 3: |  |  |  |
| Antibiotics (IV) | 1: 2: 3: |  |  |  |
| Antivirals (Oral) | 1: 2: 3: |  |  |  |
| Antivirals (IV) | 1: 2: 3: |  |  |  |
