# Supplementary material for: An international standardization to study the clinical use of lung ultrasound to discriminate viral, bacterial and atypical pneumonia in children with community acquired pneumonia
Source: Ital J Pediatr. 2025 Sep 24;51:269. doi: 10.1186/s13052-025-02113-5 (PMC12462357; doi:10.1186/s13052-025-02113-5)
Supplement: Supplementary file 2 — Supplementary Material 2 [file 13052_2025_2113_MOESM2_ESM.docx]

Dear Editor,

thank you very much for your letter of reply.

We have read the reviewers’ comments and we revised our manuscript in accordance with their suggestions, hoping we did them in the most proper way.

This is a point-by-point list of replies to each reviewer’s queries and changes made to our paper.

**Reviewer**
Reviewer reports:

Reviewer #1: The goal of this paper is to standardize the use of lung ultrasound (LUS) in diagnosing pediatric community-acquired pneumonia (CAP), but the authors do not present a clear hypothesis or specific research questions.

Thank you very much for your comment. We specified our clear hypothesis in the following paragraph: “The aim of this study is to propose a standardized protocol for diagnosing pediatric CAP. This protocol focuses on identifying distinctive LUS features associated with specific etiologies of CAP in children and integrating these LUS findings with clinical and laboratory data. The goal is to enhance the accuracy of etiological diagnosis and to guide tailored treatment strategies. Accordingly, this research seeks to develop a predictive model that combines LUS, clinical, and laboratory data to reliably differentiate among various etiologies of pediatric CAP. Furthermore, the study aims to establish a uniform model that can be adopted across multiple centers to ensure a consistent and standardized diagnostic approach.”

The paper does not explain how LUS differentiates pneumonia caused by different pathogens, nor does it compare the differences between LUS and other diagnostic methods, such as chest X-ray (CXR).

Thank you very much for your comment. We summarized the actual evidence on how LUS differentiates pneumonia caused by different pathogens in this paragraph: “Early studies identified specific LUS patterns useful for diagnosing viral lower respiratory tract infections and bronchiolitis in children (18-22). Recent works from the literature suggested LUS features can be used to determine the etiological cause of pediatric CAP (23). Berce evaluated 147 children hospitalized because of CAP, showing that LUS-detected consolidations in viral CAP were significantly smaller, with a median diameter of 15 mm, compared to 20 mm in atypical bacterial CAP(p=0.05) and 30 mm in bacterial CAP(p<0.001) (24). Buonsenso et al. showed that specific LUS patterns on diagnosis and after 48 hours of treatments (bronchograms, consolidation size, characteristics of pleural effusion) were predictive of main aetiologic groups and of antibiotic response in children with CAP, more than clinical and laboratory data (25).” As requested, we have further added more studies investigating the LUS features associated with etiological diagnosis. We have already noted that LUS demonstrates not inferior diagnostic performance compared to chest X-ray in pediatric CAP. As you suggested, we've included two paragraphs that compare LUS to both CXR and CT scan: “LUS has demonstrated higher sensitivity (up to 97%) and comparable specificity for detecting CAP when compared to CXR, particularly excelling in the identification of small, subpleural consolidations and pleural effusions that may be missed by radiography (13). Furthermore, LUS can be safely repeated for monitoring disease progression or response to therapy, which is valuable for ongoing clinical management.” and “While chest computed tomography (CT) is regarded as the gold standard for identifying pulmonary lesions in pediatric CAP, attributable to its high sensitivity (frequently above 95%) and capacity to characterize intricate findings like parenchymal necrosis, cavitation, and subtle consolidations, it is typically reserved for complicated or ambiguous cases, rather than serving as a first-line diagnostic tool, owing to considerable concerns about radiation exposure in children. Its use is quite limited in pediatric patients, as well as in other infectious conditions.”

The paper also lacks some critical elements, such as sufficient explanation of the data analysis methods, how to assess the diagnostic accuracy of LUS, and how to control for variables.

Thank you very much for your valuable comment. We have expanded and strengthened the paragraph related to the statistical analysis by inserting this: “Sources of methodological bias and confounding will be systematically identified and addressed. Operator dependency, patient-related factors (i.e., body habitus and cooperation) and prior antibiotic use will be recorded and considered as possible confounders. When feasible, analyses will be stratified according to these variables to explore their impact on diagnostic accuracy. Sensitivity analyses will test the robustness of findings after excluding cases with missing or ambiguous reference standard diagnosis. Where center variability may influence results, mixed-effects or hierarchical models will be employed. Any residual confounding or technical limitations impacting LUS interpretation will be transparently reported and discussed.”.

Moreover, it does not mention how to ensure the standardization of operations across participating centers or operators.
Thank you very much for your comment. We specified it in the following paragraph: “To ensure the reliability and reproducibility of LUS findings across all participating centers, rigorous standardization measures will be implemented. First, all operators will undergo a dedicated training program based on internationally recognized LUS protocols, including both theoretical instruction and hands-on supervised practice. Certification of proficiency will be required prior to independent scanning, and periodic refresher sessions will be organized to maintain high standards of performance. This detailed, standardized scanning protocol will be distributed to all centers, specifying probe selection, patient positioning, scanning windows, and image acquisition parameters. To minimize inter-operator variability, all LUS examinations will be performed using a predefined set of scanning planes and documented using a uniform image storage and labeling system, as detailed below. Centralized quality control will be established: a proportion of LUS images and clips from each center will be reviewed by an expert panel blinded to clinical data, ensuring adherence to protocol and consistency in interpretation. Additionally, all data will be entered into a centralized electronic database with built-in checks for completeness and consistency.”.

The authors should cite relevant literature and discuss the advantages and disadvantages of LUS compared to traditional diagnostic methods (such as chest X-ray, PCR, etc.) to enhance the credibility of the research.

Thank you very much for your comment. We added more details and data about comparison with other techniques, as specified above.

It is recommended that the authors provide more details in the ethics section, particularly regarding specific measures for participant selection and data protection.
Thank you very much for your valuable observation. We added the following paragraph: “Participant selection will be conducted strictly according to the predefined inclusion and exclusion criteria to ensure the scientific validity and ethical integrity of the study. Informed consent will be obtained from parents or legal guardians prior to any study-related procedures, and assent will be sought from children as appropriate for their age and level of understanding, in accordance with the Declaration of Helsinki and local regulations. To ensure robust data protection, all personal and health information will be handled in compliance with the European General Data Protection Regulation (GDPR) and relevant national laws. Each participant will be assigned a unique study identification code, and all data will be pseudonymized prior to analysis. Identifiable information will be accessible only to authorized study personnel at each site and will be stored on secure, password-protected servers. Data transfer between centers will use encrypted channels, and only de-identified datasets will be shared for central analysis. Access to the electronic database will be restricted to designated investigators, with audit trails maintained to monitor data access and modifications. All study documents and source data will be retained for the period required by regulatory authorities. Participants and their families will be informed of their rights regarding data access, correction, and withdrawal from the study at any time without consequences for their medical care.”

The authors should clearly explain how data standardization will be ensured between centers, especially concerning the use of electronic health records (EHR) and the storage of LUS images.

We added the following paragraph: “**Data standardization across different centers**

To ensure data standardization across all participating centers, a unified electronic data capture (EDC) system will be implemented for clinical, laboratory, and imaging data. All centers will utilize a standardized EHR template, showed in **Supplementary Materials**, which defines required data fields, permissible values, and standardized terminology for clinical variables. This EHR template will be distributed to all sites and training will be provided to ensure consistent data entry practices. For LUS image storage, all centers will follow a harmonized protocol specifying image acquisition parameters, file formats (e.g., DICOM), and mandatory metadata (e.g., patient study ID, date, and anatomical site). LUS images and video clips will be uploaded to a secure, centralized, cloud-based repository with restricted access, allowing for real-time quality control and centralized review by an expert panel.”

The authors should detail how the study results will be presented and analyzed, how the diagnostic accuracy of LUS will be quantified, and whether they will assess sensitivity, specificity, ROC curves, or use other methods.
Thank you very much for your comment. We illustrated how the predictive models will be progressively structured in statistical analysis section. We further added more details in this paragraph: “Model calibration will be assessed using the Hosmer-Lemeshow goodness-of-fit test, and multicollinearity among predictors will be evaluated using the variance inflation factor. Effect size estimates will be reported as odds ratios with corresponding 95% confidence intervals. Sensitivity analyses will be conducted to assess the robustness of findings, including evaluating models in key subgroups and, where appropriate, using alternative CRP cut-off thresholds or excluding cases with missing data.”.
The paper mentions global collaboration but does not specify how this will be achieved, particularly in terms of data sharing, establishing an international network, and the role of AI in future research.

Our goal is to establish a collaborative scientific initiative. By using the aforementioned standardized methods, we aim to create a comprehensive, shared dataset that integrates data from various centers. This will enable us to manage and analyze a vast amount of data using state-of-the-art AI techniques, such as deep learning, to better understand and predict outcomes.

The paper does not adequately discuss the limitations of LUS as a diagnostic tool, especially concerning technical constraints, operator dependency, or patient factors (such as obesity or the inability to remain still).

Thank you very much for your comment. We widely discussed them in the following paragraph: “Despite its proven high accuracy, LUS as a diagnostic tool for pediatric CAP faces several limitations. A primary concern is operator dependency, where the accuracy of LUS is heavily dependent on the sonographer's expertise and experience. Variations in interpretation, especially by less skilled personnel lacking standardized training, can lead to misdiagnosis. Technical constraints related to ultrasound equipment also impact image quality and diagnostic reliability. Factors such as probe type selection, time gain compensation, and beam focusing can lead to misdiagnosis of pulmonary consolidations. Patient-related factors are furtherly challenging. Obesity, in particular, can impair ultrasound wave penetration due to increased chest wall thickness, thereby reducing image clarity and diagnostic yield. Furthermore, the inability of some pediatric patients to remain still during the examination can hinder image acquisition and prolong the procedure, potentially compromising diagnostic accuracy. Moreover, LUS has a limited ability to detect lesions not in direct contact with the pleura or located in lung areas difficult to access by ultrasound, such as deep parenchymal lesions, which may result in false negatives.”.


Reviewer #2: The part on the description of LUS is long and in-depth,
perhaps to be streamlined and shortened.

Thank you very much for your comment. We have revised the first part as per your suggestion and have removed the pleonastic details accordingly.
The second part on etiological research is instead a little more superficial;
more details on the methods that will be used in practice would be useful.

Thank you very much for your comment. In response to your request, we have added additional details.
The conclusions are fine.

Thank you very much for your comment.

I hope that after these corrections the manuscript is improved and it could have, in case of publication, a good reception and a good impact by readers.

Best regards,

Danilo Buonsenso, on behalf of co-authors
